# Regulation of angiogenesis and invasion by human Pituitary tumor transforming gene (PTTG) through increased expression and secretion of matrix metalloproteinase-2 (MMP-2)

**DOI:** 10.1186/1476-4598-5-61

**Published:** 2006-11-10

**Authors:** Mohammad T Malik, Sham S Kakar

**Affiliations:** 1 Department of Biochemistry and Molecular Biology, University of Louisville, Louisville, KY, USA; 2 Department of Medicine and James Graham Brown cancer Center, University of Louisville, Louisville, KY, USA

## Abstract

**Background:**

Pituitary tumor transforming gene (PTTG) is a novel oncogene that is expressed at higher level in most of the tumors analyzed to date compared to normal tissues. Existence of a relationship between PTTG levels and tumor angiogenesis and metastasis has been reported. However, the mechanisms by which PTTG achieve these functions remain unknown. In the present study, we investigated the effect of overexpression of PTTG on secretion and expression of metastasis-related metalloproteinase-2 (MMP-2) in HEK293 cells, cell migration, invasion and tubule formation.

**Results:**

Transient or stable transfection of HEK293 cells with PTTG cDNA showed a significant increase in secretion and expression of MMP-2 measured by zymography, reverse transcriptase (RT/PCR), ELISA, and MMP-2 gene promoter activity. Furthermore, in our studies, we showed that tumor developed in nude mice on injection of HEK293 cells that constitutively express PTTG expressed high levels of both MMP-2 mRNA and protein, and MMP-2 activity. Conditioned medium collected from the HEK293 cells overexpressing PTTG showed a significant increase in cell migration, invasion and tubule formation of human umbilical vein endothelial cells (HUVEC). Pretreatment of conditioned medium with MMP-2-specific antibody significantly decreased these effects, suggesting that PTTG may contribute to tumor angiogenesis and metastasis via activation of proteolysis and increase in invasion through modulation of MMP-2 activity and expression.

**Conclusion:**

Our results provide novel information that PTTG contributes to cell migration, invasion and angiogenesis by induction of MMP-2 secretion and expression. Furthermore, we showed that tumors developed in nude mice on injection of HEK293 cells that constitutively express PTTG induce expression of MMP-2 and significantly increase its functional activity, suggesting a relationship between PTTG levels and MMP-2 which may play a critical role in regulation of tumor growth, angiogenesis and metastasis. Blocking of function of PTTG or down regulation of its expression in tumors may result in suppression of tumor growth and metastasis, through the down regulation of MMP-2 expression and activity. To our knowledge, this study is the first study demonstrating the modulation of MMP-2 expression and biological activity by PTTG.

## Background

Primary tumor growth is restricted due to limited supply of oxygen, nutrients, and growth factors. Tumor progression and invasion to distant organs depends on tumor angiogenesis. To achieve angiogenesis various growth and transforming genes are turned on to enable tumor progression [1]. Once angiogenesis is initiated, the tumor expands exponentially and invades to local and distance tissues. From a patient survival perspective point of view, understanding the mechanisms of angiogenesis and metastasis for the development of new therapeutics is a critical step to treat tumors [2,3].

In this contest, many oncogenes have been reported to play important roles in tumor angiogenesis and metastasis. Recently, a novel oncogene, pituitary tumor transforming gene (PTTG), also known as securin, has been reported to play a vital role in tumor angiogenesis [4]. Using an mRNA differential technique, PTTG was originally cloned from a rat pituitary tumor [5], followed by cloning of its homologue from humans [6-8]. The predominant cellular location of the PTTG protein is the cytoplasm, although it is partially localized in the nucleus [9]. Nuclear translocation of PTTG can be facilitated by either interaction with PTTG binding factor (PBF) [10], or by interaction with the mitogen-activating protein (MAP) kinase cascade [11]. The level of PTTG expression is increased in rapidly proliferating cells and is regulated in a cell cycle-dependent manner [9,12]. PTTG expression is low at the G1/S interphase, increases gradually during the S phase, and peaks at the G2/M phase [12]. As the cells enter into anaphase, PTTG is degraded and daughter cells express very low amounts of PTTG. The degradation of PTTG most likely occurs via ubiquitination since PTTG contains a D box which is required for such proteolysis [9].

Numerous studies have demonstrated that human PTTG displays a distinct pattern of expression. In normal tissues, PTTG expression is restricted, with high levels in testis, and low levels in the thymus, colon, and small intestine [6,13]. In contrast, PTTG is highly expressed in a variety of human primary tumors as well as tumor cell lines including tumor of the ovary, lung, testis, kidney, colon, thyroid, pituitary, liver, adrenal, breast, prostate, melanoma, leukemia, and lymphoma [6-9,12-19], suggesting that PTTG may be involved in tumorigenesis. Furthermore, increase in expression levels of PTTG have been predicted to correlate with increased tumor invasiveness in human pituitary tumors with hormone overproduction [16], and with the degree of malignancy, pathogenesis and/or progression of colorectal and thyroid tumors [14,20]. PTTG has been identified as one of eight signature genes that is associated with tumor metastasis andis up regulated in human primary solid tumors [15]. A relationship between the survival rate and level of expression of PTTG in esophageal cancer has been reported [21].

We and others have shown that overexpression of human PTTG in mouse fibroblasts (NIH3T3) and human embryonic kidney (HEK293) cells increases cell proliferation, induces cellular transformation *in vitro *and promotes tumor formation in nude mice [5,6,22]. Currently, the precise mechanism by which PTTG causes cell transformation remains unclear. Data from our laboratory and others suggest that PTTG may act through basic growth factor (bFGF) [22,23], vascular endothelial growth factor (VEGF) [4,22], and/or interleukin-8 (IL-8) [22]. Additional mechanism by which PTTG may induce its oncogenic function is indicated by findings that implicates its role in sister chromatid separation during cell division [24]. PTTG, by virtue of its function as a human securin, ensures that there is no premature separation of sister chromatids. Mice lacking PTTG show aberrant cell cycle progression, premature centromere division, and problems with chromosomal stability, as well as tissue specific phenotypes, such as testicular and splenic hypoplasia and thymic hyperplasia[25]. In addition, PTTG null mice exhibit impaired proliferation of pancreatic beta cells and develop type I diabetes during late adulthood [26]. Furthermore, crossbreeding of PTTG (-/-) mice with Rb (+/-) mice showed significant reduction in pituitary tumor development compared to PTTG (+/+)/Rb (+/-) mice; incidence of pituitary development decreased from 80% in PTTG (+/+)/Rb (+/-) animals to 30% in PTTG (-/-)/Rb (+/-) animals [27]. On the other hand, transgenic animals that express PTTG under the control of the αGSU promoter showed enlarged pituitary and hyperplasia of prostate [28]. Taken together, these data strongly suggest an important role of PTTG in cell proliferation and tumorigenesis.

Local invasive growth is a key feature of primary malignant tumor. A correlation between the level of expression of PTTG and invasiveness and degree of malignancy in pituitary and colorectoral tumors has been reported [14]. However, the specific mechanisms by which PTTG facilitates invasive behavior remain unknown. It is known that interactions between cancer cells and surrounding normal cells and the extra-cellular matrix (ECM) are the key events in tumor cell invasion [29,30]. For successful invasion and to spread tumor cells through the surrounding normal tissue, tumor cells must degrade multiple elements of the ECM, including fibronectin, laminin, and type IV collagen [31,32]. Metalloproteinases (MMPs) are potent proteolytic enzymes known to play key roles in degradation of basement membranes and extracellular matrix required for tumor cells to invade and metastasize. The MMP family contains 24 members, of which MMP-2 (gelatinase A, 72-kDa type IV collagenase) and gelatinase B (92-kDa type IV collagenase) have been observed in several cancers including breast cancer, colon cancer, skin cancer and lung cancer [33-37] and their expression has been correlated with local invasion by the tumor, lymph node metastasis and survival rate [31-34,38,39]. Both MMP-2 and MMP-9 cleave the type IV collagen and gelatin, which are the principal structural components of the basement membrane (40). Since, PTTG is over-expressed in most of tumors and its role in angiogenesis and metastasis has been reported [14,15,20], we hypothesize that some of the functions of PTTG may be mediated through the regulation of expression of MMP-2 metalloproteinase and modulation of its biological activity. In the present study, we show that overexpression of PTTG in HEK293 cells results in up-regulation of secretion and expression of MMP-2, but not MMP-9, resulting in increased cell migration and invasion.

## Results

### PTTG increases the expression and activity of MMP-2 *in-vitro*

**Figure 1:**
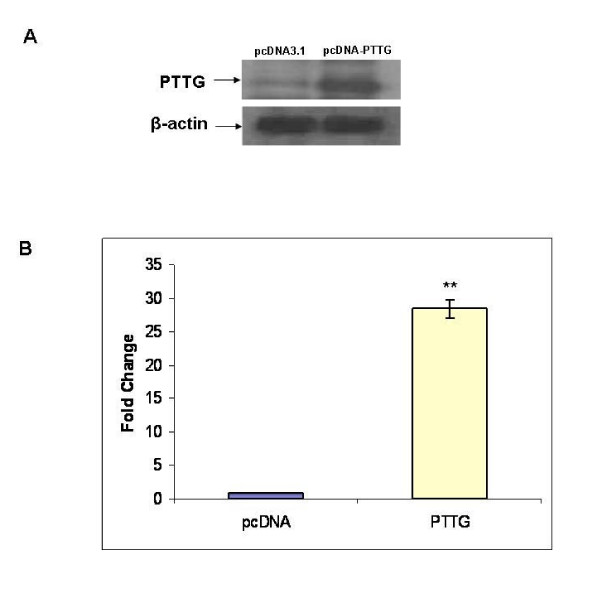
Western blot analysis of PTTG. A: HEK293 cells were transiently transfected with PTTG cDNA or pcDNA3.1 vector. After 48 h of transfection, cell lysates were prepared and used for Western blot analysis using the PTTG-specific antiserum. The blot was stripped and used for β-actin to examine equal loading of proteins. B: Graphical representation of Data shown in A. The immuno-reactive bands were scanned with densitometer, quantitated and normalized with β-actin. The data shown represent mean ± SEM of three independent experiments. ** = p < 0.05.

**Figure 2:**
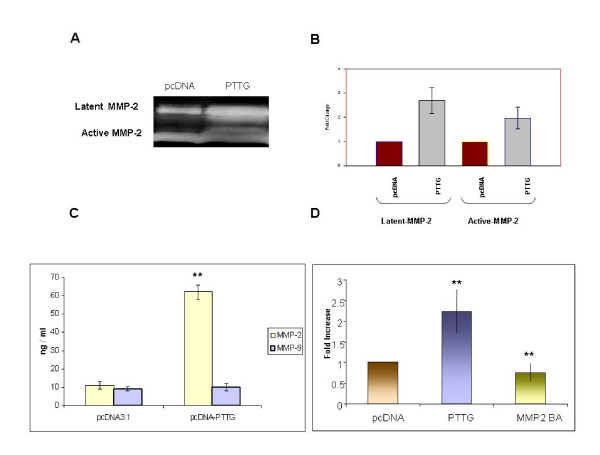
MMP-2 secretion and expression. A: Zymography analysis. Conditioned medium from HEK293 cells transiently or stably transfected with PTTG cDNA or pcDNA3.1 vector was collected. MMP-2 and MMP-9 levels in the medium were determined by gelatin zymography. Both active (68 kDa) and latent (72 kDa) forms of MMP-2 are shown. B: Graphical representation of the data shown in A. Data represents mean ± SEM from three independent experiments. C: ELISA assay. Levels of MMP-2 and MMP-9 (ng/ml) in conditioned medium collected from HEK293 cells transfected with PTTG cDNA or pcDNA3.1 vector. Data represent mean ± SEM from three independent experiments. ** = p < 0.05. D: Measurement of MMP-2 functional activity using Direct Quenching (DQ) fluorescence conjugate collagenase analysis. DQ assay was performed as described in materials and methods. Activity of collagenase was approximately 2.5 fold higher in CM from HEK293 cells transfected with PTTG cDNA as compared to CM collected from cells transfected with pcDNA3.1 vector. Addition of MMP-2 blocking antibody (MMP2BA) completely blocked the collagenase activity. Data represent the mean ± SEM of three independent experiments performed in triplicate. ** = p < 0.05.

Levels of both PTTG and MMP-2 are reported to correlate with the angiogenesis and metastasis of tumor[15,35-38], suggesting for a existence of relationship between PTTG and MMP-2. To examine this possibility, we examine the secretion and expression of MMP-2 in HEK293 cells transiently or stably transfected with PTTG cDNA. As shown in Fig. 1, HEK293 cells showed very low level of expression of PTTG protein; however, transient transfection of these cells with PTTG cDNA resulted in a significant increase in expression of PTTG protein. Similar results were obtained when HEK293 cells were stably transfected with PTTG cDNA (results not shown). To examine whether PTTG modulate the secretion of MMP-2 and MMP-9 from HEK293 cells, we determined the MMP-2 and MMP-9 levels in the conditioned medium by zymography. As shown in Fig. 2A and 2C, transfection of HEK293 cells with PTTG cDNA resulted in enhanced secretion of MMP-2 (~2-3-fold) but not MMP-9 secretion (results not shown). These results were further confirmed by ELISA, which showed ~5-fold increase in MMP-2 levels in CM collected from HEK293 cells compared to CM collected from cells transfected with pcDNA3.1 vector (Fig. 2C). Furthermore, PTTG was found to increase the levels of both forms of MMP-2 (active 62 kDa; and latent 72 kDa) (Fig. 2A and 2B). Addition of MMP-2-specific antibody to the conditioned medium significantly decreased the activity of MMP-2 (digestion of fluorescence collagenase substrate) (Fig. 2D).

**Figure 3:**
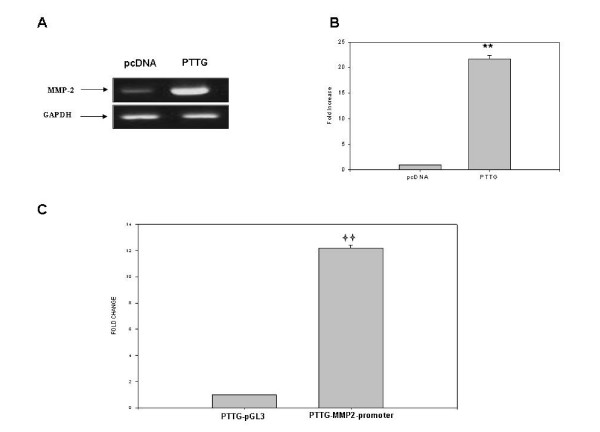
Expression of MMP-2. A: Semi quantitative RT/PCR analysis of MMP-2 mRNA in HEK293 cells transfected with PTTG CDNA or pcDNA3.1 vector. GAPDH was used as an internal control. B: PCR products shown in A were scanned and quantitated. MMP-2 expression was increased by ~22 fold in HEK293 cells transfected with PTTG cDNA compared to cells transfected with pcDNA3.1. C: Measurement of MMP-2 gene promoter activity. HEK293 cells were co-transfected with MMP-2 gene promoter and PTTG cDNA or pcDNA3.1 vector. MMP-2 promoter gene promoter activity was measured by assaying luciferase activity. The data shown represent fold change of MMP-2 promoter activity in HEK293 cells when co-transfected with pGL2-MMP-2 and PTTG cDNA compared to cells co-transfected with pGL2-MMP-2 and pcDNA3.1 vector. Values were normalized with renila luciferase activity used as an internal control and represent as mean ± SEM of three independent experiments performed in triplicate. ** = p < 0.05.

Increase in secretion of MMP-2 in CM by PTTG could be result of increase in translation of MMP-2 protein from MMP-2 mRNA or induction of secretion from stored vesicles. To elucidate whether PTTG-induced MMP-2 secretion was transcriptionally controlled, mRNA expression levels of MPP-2 were examined by semi-quantitative RT/PCR. Transfection of HEK293 cells with PTTG cDNA resulted in a significant up-regulation of MMP-2 mRNA compared to cells transfected with pcDNA3.1 vector (Fig. 3A and 3B), suggesting that enhanced MMP-2 secretion by PTTG may be regulated at the transcriptional level. Further to confirm that increase in MMP-2 mRNA levels were in fact due to change in transcriptional activity of MMP-2 gene and not due to change in degradation or stability of MMP-2 mRNA; we determined MMP-2 gene promoter activity. As shown in Fig. 3C, our data demonstrated a significant (~11-fold) increase in MMP-2 gene promoter activity in cells transfected with PPTG cDNA compared to cells transfected with pcDNA3.1 vector. Collectively, our data suggest that PTTG increases MMP-2 secretion and expression in HEK293 cells, and such increase by PTTG at least in part is due to activation of MMP-2 gene transcription.

### PTTG increases invasion of HUVEC *in vitro*

**Figure 4:**
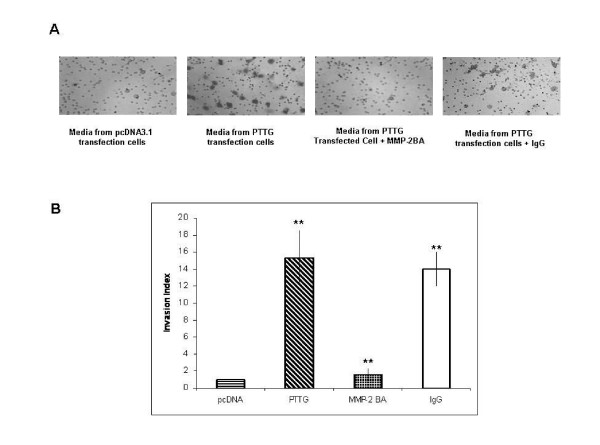
Cell invasion assay. HUVEC were transferred to upper chamber. CM collected from HEK293 cells transfected with PTTG cDNA or pcDNA3.1 vector was transferred to the lower chamber. Cell invasion index was calculated as described in Methods. CM was pre-incubated with MMP-2-specific antibody or control mouse IgG. B: Graphical representation of the data shown in A. Data represent mean ± SEM of three independent experiments performed in triplicate. ** = p < 0.05.

To determine the role of PTTG on invasion and migration of tumor cells through MMP-2, we used stably transfected HEK293 cells. HUVEC suspended in serum free medium were transferred to upper chamber of Boyden wells and CM collected from HEK293 cells constitutively expressing PTTG or pcDNA3.1 vector transfected cells was added to the lower chamber as described in Materials and Methods. Cells showed higher invasive index when CM from HEK293 cells constitutively expressing PTTG was used compared to CM from HEK293 cells stably transfected with vector (Fig. 4). Such increase in cell invasion was completely blocked by addition of MMP-2-specific antibody (2 μg/ml) to the medium but not by adding equal amounts of control mouse IgG, suggesting that increase of invasion of cells was due to increased MMP-2 secretion from HEK293 cells as a result of overexpression of PTTG.

### PTTG increases the migration of HUVEC *in vitro*

**Figure 5:**
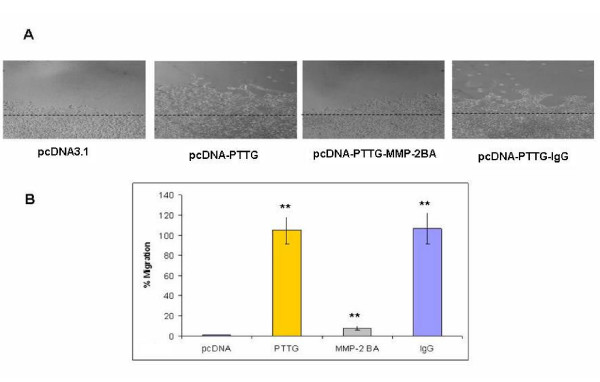
Cell migration. Medium from HEK293 cells transfected with PTTG cDNA or pcDNA3.1 was used for HUVEC cells plated in 35 mm dishes. A: Wound assay showing the migration of cells in wounded area. B: Graphical representation of data shown in A. A significant increase in HUVEC migration was observed when CM from HEK293 cells transfected with PTTG cDNA was used compared to CM from cell transfected with pcDNA3.1 vector. Addition of MMP-2-specific antibody significantly decreased the cell migration, whereas addition of control mouse IgG to CM was ineffective. Data shown represent mean ± SEM from three independent experiments performed in triplicate. ** = p < 0.05.

To determine the effect of PTTG on cell migration, we performed wound migration assays in 35 mm culture plates. HUVEC cells were plated in 35 mm dishes and wound was created as described in Materials and Methods. Cells were incubated for 48 h in CM collected from HEK293 cells stably transfected with PTTG cDNA or pcDNA3.1 vector. Cell migration was quantified by counting the number of cells that migrated into the wounded area with a microscope fitted with an eyepiece marked grid (1 mm^2^). As shown in Fig. 5, there was a 95% increase in cell number migrated into wounded area when CM collected from PTTG cDNA transfected cells was used compared the CM collected from cells transfected with pcDNA3.1 vector. Addition of MMP-2-specific antibody to CM collected from HEK293 cells transfected with PTTG cDNA resulted a significant (87%) decreased in cell migration, whereas addition of control mouse IgG showed no inhibitory effect, suggesting an important role of MMP-2 in cell migration.

### PTTG increases tubule formation and growth in 3D Matrigel matrix

**Figure 6:**
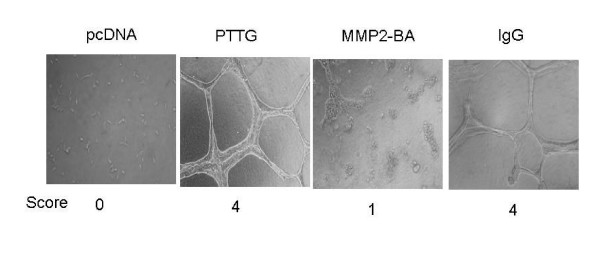
Tubule formation assay: HUVEC cells were grown in Matrigel matrix mixed with CM collected from HEK293 cells transfected with PTTG cDNA (PTTG) or pcDNA3.1 vector (pcDNA). MMP-2-specific antibody was added to CM collected from HEK293 transfected with PTTG cDNA (MMP2-BA) or control mouse IgG (IgG).

MMP-2 is known to play an important role in the proteolysis of ECM which allows endothelial cells to migrate towards the angiogenic stimuli to form new blood vessels to nurture tumor cells to grow. To determine if PTTG plays a role in regulating MMP-2 function in such process, we suspended HUVEC in CM collected from HEK293 cells stably transfected with PTTG cDNA or pcDNA3.1 vector and mixed with Matrigel matrix and assayed for the tubule formation as described in materials and methods. As shown in Fig. 6, cells suspended in CM collected from HEK293 cells transfected with PTTG cDNA formed well defined tubules, whereas cells suspended in CM collected from HEK293 cells transfected with pcDNA3.1 vector did not form tubules or formed undefined and irregular tubules. Again addition of MMP-2 blocking antibody to the CM collected from HEK293 cells with PTTG cDNA completely abrogated tubule formation. On the other hand addition of control mouse IgG to the CM was ineffective, suggesting that increased in MMP-2 secretion in CM is due to overexpression of PTTG in HEK293 cells is essential for tubule formation.

### PTTG increases the secretion and expression of MMP-2 *in vivo*

**Figure 7:**
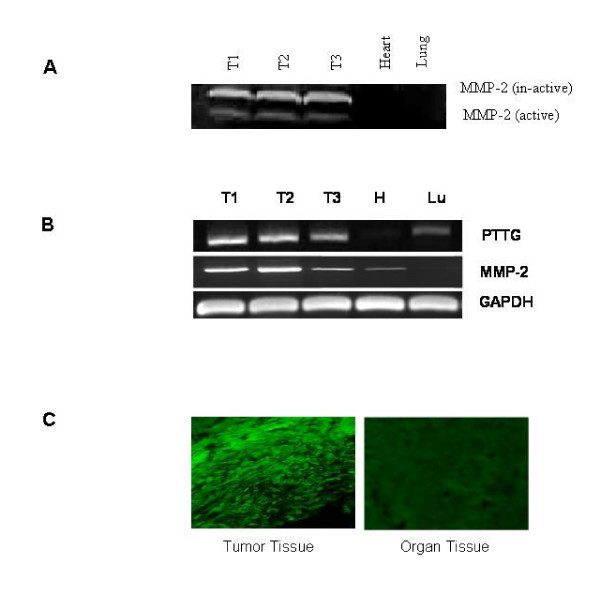
Expression of PTTG and MMP-2 in tumors and normal tissues. Tumors and other tissues were collected from nu/nu mice that developed tumors on injection of HEK293 cells constitutively expressing PTTG. A: Zymography analysis of tumor and normal tissues lysates. B: Semi-quantitative RT/PCR analysis of PTTG and MMP-2 expression using specific primers for PTTG and MMP-2 genes. GAPDH was used as an internal control. C: MMP-2 functional *in situ *fluorescence collagenase activity assay. The data shown are representative of three independent experiments.

As reported above, increase in PTTG expression activates secretion and expression of MMP-2 in vitro, we were interested to examine if similar effects of PTTG are sustained in vivo. To test it, we collected the tumors developed in nude mice on injection of HEK293 cells constitutively expressing PTTG as described previously [22]. Analysis of tumors for MMP-2 secretion, expression and MMP-2 functional activity revealed a significant increase in secretion of MMP-2 in tumors (measured by zymography), MMP-2 mRNA levels (measured by semi-quantitative RT/PCR) and MMP-2 functional activity (measured by DQ fluorescence substrate activity) (Fig. 7) compared to other tissues collected from the same animals. HEK293 cells transfected with pcDNA3.1 vector or untransfected HEK293 cells did not develop tumors or developed very small tumors [22], therefore were not available for comparative analysis.

## Discussion

Pituitary tumor transforming gene (PTTG) is a potent oncogene. It is expressed at high levels in almost all the tumors analyzed to date. Its oncogenic potential was demonstrated by its overexpression in mouse fibroblast NIH3T3 cells and human embryonic kidney HEK293 cells, which showed increased cell proliferation, colony formation on soft agar and tumor formation in nude mice [5,6,13,22]. High levels of PTTG expression have been reported to correlate with highly aggressive and metastatic tumors [15,41], and to regulate the expression of many growth and angiogenic factors including bFGF, VEGF and IL-8 [4,22,23,41]. However, the mechanisms by which PTTG mediate its angiogenic and metastatic effects remain unclear. Metalloproteinases are central to the ability of cancer cells that play important role in tumor angiogenesis and metastasis by regulating the digestion of extracellular matrix (ECM) to make way for tumor cells to move and invade. It is well recognized that MMPs are also involved in the expression and activation of various chemokine, growth factors and their receptors [43] required for biological and pathological processes including angiogenesis and tumor growth [42,44,45]. Multiple MMPs are involved in the degradation of the surrounding matrix, but MMP-2 has been at the forefront in tumor invasion, angiogenesis, and metastases. Tumor and stromal cells express high levels of MMP-2, which allows the cells to invade and metastasize [29,30]. Importance of MMP-2 in tumor progression and angiogenesis was investigated by Itoh et al. [46] who using MMP-2 deficient mice showed reduced tumor burden and angiogenesis in these mice. On the other hand Kato et al. [47] reported decreased in the bFGF-induced corneal neovascularization in MMP-2 deficient mice accompanied by reduction in vascular endothelial cell migration and tubules formation.

Based on this information, we hypothesize that there exists a relationship between PTTG and MMP-2 and some of the oncogenic functions of PTTG may be mediated through the modulation of expression and activity of MMP-2. In our studies, we clearly showed that overexpression of PTTG in HEK293 cells resulted in a significant increase in secretion of MMP-2 (Fig. 2) in the medium and its mRNA levels in cells expressing PTTG compared to cells transfected with pcDNA3.1 vector (Fig. 3). Such increase in expression of MMP-2 by PTTG was found to be at least in part through the regulation of MMP-2 gene transcription.

Increase in MMP-2 levels showed increase in cell migration, invasion and tubule formation of HUVEC when exposed to CM collected from cells transfected with PTTG cDNA. Such effects of MMP-2 were completely blocked when MMP-2-specific antibody were included in the medium, specifically confirming the effects of MMP-2 in cell migration, invasion and angiogenesis (Figs. 4, 5, 6). PTTG is know to increase tumor angiogenesis and its relationship with tumor metastasis has been reported [4,15]. Our results clearly demonstrate that PTTG increases the secretion and expression of MMP-2 which in turn regulates cell migration, invasion and tubule formation in vitro. Therefore, it is plausible that PTTG may serve as one of the major regulators of MMP-2 and some of the oncogenic effects of PTTG are being mediated through the regulation of expression of MMP-2.

Membrane type-1 (MT1) MMP specifically activates the pro-gelatinase, MMP-2 on tumor cell surface in vitro and binds with tissue inhibitor of matrixproteinases (TIMPs), hence, controlling MMP-2 activity [48]. The delicate balance between MMP and TIMP levels ultimately determines the functions of MMP by protein degradation in vitro [48]. Most of the MMP expression induced by activated MMP accompanies increased expression of one of the TIMPs as a part of defense mechanism. TIMP2 protein inhibits the activation of proMMP2 by forming a stable complex with activated MT1-MMP, whereas low concentration of TIMP2 can form a tertiary complex with MT1-MMP and proMMP2 to eventually activate MMP2. Thus, MT1-MMP combines with TIMP2 and influences the activation state of MMP2. In our studies we observed a significant increase in levels of MT1-MMP mRNA in HEK293 cells when transfected with PTTG cDNA (data not shown), suggesting regulation of expression of both MMP-2 and MT1-MMP by PTTG to achieve the required activation of MMP-2 to mediate its tumorigenic function. In our studies, we also demonstrated that PTTG not only regulates the expression of MMP-2 *in vitro *but it does regulate MMP-2 expression and function *in vivo *(Fig. 7), however, it remains unclear if blocking of MMP-2 activity *in vivo *will inhibit angiogenesis and tumor development as result of overexpression of PTTG in tumors

## Methods

### Cell culture

Human embryonic kidney cells (HEK293) and Human umbilical vein endothelial cells (HUVEC) were obtained form American Type Culture Collection (ATCC) (Manassas, VA) and were maintained at 37°C in an atmosphere of 5% CO_2 _in humidified air. HEK293 cells were cultured in DMEM (Invitrogen, Carlsbad, CA) supplemented with penicillin/streptomycin (100 IU/ml and 100 μg/ml) and 10% fetal bovine serum (FCS) (Hyclone, Atlanta, GA). HUVEC cells were cultured in ECM medium (Clonetics, Walkersville, MD) supplemented with Bullet Kit (EGM-2). The cell lines were subcultured on routine basis every 3–4 days. The MMP-2 blocking antibody (MMP2BA) was obtained from Chemicon (Temecula, CA). Porcine gelatin and clostridium collagenases were from Sigma Chemical Co. (St. Louis, MO). DQ collagen fluorescein conjugate was purchased from Molecular Probes (Carlsbad, CA).

### Transfection and collection of conditioned medium (CM)

For transient transfection, HEK293 cells were plated into six well plates approximately 24 h prior to transfection. Cells were transfected in serum-free medium using 1 μg of plasmid DNA and 3 μl of transfectin (BioRad, Hercules, CA) according to the manufacture's instructions. After 18 h of transfection, medium was replaced with serum-free medium (DMEM) (Invitrogen) ± MMP-2 blocking antibody 2 μg/ml. After 48 h, conditioned medium (CM) was collected, centrifuged and stored at -80°C. Tissue lysates and stable clones of HEK293 cells transfected with pcDNA3.1 or PTTG cDNA were generated as describe previously [22].

### Western blot analysis

For western blot analysis, cells were lysed with lysis buffer (20 mM Tris HCl pH7.5, 1 mM EDTA, 0.1% Nonidet P-40, 1 mM PMSF, and 1μg/ml each of pepstatin, leupeptin and aprotinin). Protein concentration for each sample was determined by Bradford method (Bio-Rad). An equal amount of protein (40 μg) from each sample was subjected to SDS-PAGE as described previously [49]. The proteins were blotted onto nitrocellulose membrane and was incubated with PTTG antiserum diluted at 1:1,500 at room temperature for 1 h, followed by incubation with goat anti-rabbit HRP-conjugated PTTG secondary antibody for 45 min. Immuno-reactive proteins were detected by using enhanced chemiluminescent (ECL) assay system from GE Healthcare Biosciences, Corp. (Piscataway, NJ).

### Measurement of secretion of MMP-2 and MMP-9 in conditioned medium

#### A: ELISA assays

To determine the effect of PTTG on secretion of MMP-2 and MMP-9 in tissue culture medium, we measured the levels of MMP-2 and MMP-9 in CM collected from HEK293 cells transfected with PTTG cDNA using commercially available ELISA kits from BD Biosciences (Minneapolis, MN) and zymography. Briefly, HEK293 cells were transiently transfected with pcDNA3.1 or pcDNA3.1-PTTG cDNA using transfectin transfection reagent (BioRad) as described above. After 24 h of transfection, the medium was replaced with serum free DMEM medium. Twenty-four h later, the medium was collected, centrifuged and concentrated by 5-fold (1.0 ml to 200 μl) using a speedVac system (Savant, Holbrook, NY). Protein was determined by the Bradford method [50]. Levels of MMP-2 and MMP-9 were determined by ELISA assays and zymography. ELISA assays were performed according to instructions from the supplier. All measurements were normalized to protein concentration and performed in triplicate.

#### B: Zymography

Gelatin zymography analysis was carried out as described by Heussen et al. [51]. Briefly 20 μg of CM protein from each sample was loaded on polyacrlyamide gel containing 0.1% gelatin (Sigma Co., St. Louis, MO). Electrophoresis was performed under non-reducing conditions at 20 mA for 3 h at room temperature. The gel was washed twice for 30 min each in 2.5% Triton X-100 to remove SDS, incubated in substrate buffer (50 mM Tris-HCl, 5 mM CaCl_2_, 0.01% NaN_3_, pH 7.6) for 24 h at 37°C. Gel was stained with 0.5% Coomassie brilliant blue G-250 (Pierce Rockford, IL) for 30 min at room temperature, and destained (30% ethanol, 10% acetic acid, 60% water). The presence of MMP-2 was indicated by an unstained proteolytic zone of substrate at position of 72 kDa (MMP-2 active form) and 68 kDa (MMP-2 latent form) compared to maker applied on the same gel). The gel was scanned using NIH image software, and data are presented as fold change compared to control i.e. pcDNA transfected conditioned medium.

### Measurement of MMP-2 activity

To determine change in MMP-2 activity 20 μl of collagen fluorescent substrate was reconstituted in PBS, mixed with 80 μl of reaction buffer (0.5 M Tris-HCl, 1.5 M NaCl, 50 mM CaCl_2_, 2 mM NaN_3_, pH 7.6) and added to the fluorescence reader plate. Twenty μl of CM treated with or without MMP-2 blocking antibody collected from HEK293 cells transfected with PTTG cDNA or pcDNA3.1 vector was added to each well. Clostridium collagenase was serially diluted and used as a positive control and reaction buffer was used as a negative control. The plate was incubated at room temperature for 1 h. After incubation, the intensity of the digested product from DQ collagen was measured at excitation 495 nm and emission at 515 nm in the fluorescence microplate reader. The fold change was calculated by dividing the mean of fluorescence values of CM collected from cells transfected with PTTG cDNA by the mean value of fluorescence of conditioned medium collected from cells transfected with pcDNA3.1 vector.

### Measurement of MMP-2 activity in tumors

MMP-2 activity and expression in tumors and normal tissues collected from *nu/nu *mice as describe previously [22]. Briefly, nu/nu mice were injected subcuatneously with HEK293 cells constitutively expressing PTTG. After four weeks of injection when large tumors were developed, animals were scarified. Tumors and other tissues (lung, heart, kidney and liver) from the same animal were collected and stored at -80°C for further use. For MMP-2 activity and expression, DQ collagen substrate (1 mg/ml) was mixed with reaction buffer (0.5 M Tris-HCl, 1.5 M NaCl, 50 mM CaCl_2_, 2 mM NaN_3_, pH 7.6) containing 0.8% low melt agarose (Invitrogen) at a ratio of 1:1 according to Gailis et al. [52]. After melting agarose mixture at 60°C, the mixture was applied on the frozen tissue sections on glass slides and allowed to solidify at room temperature. Slides were incubated in a humidified chamber with a few drops of reaction buffer on each slide for 5–7 days. Assessment of degradation of substrate was examined with an Olympus fluorescence microscope and photographed with a KODAK DC290 digital camera.

### Determination of MMP-2 mRNA levels using RT/PCR

Total RNA was isolated from transfected HEK293 cells in log phase or tumor and normal tissues using Trizol reagent (Invitrogen) as described previously [6]. First strand cDNA was synthesized using iScript RT-PCR kit (Bio-Rad) and subsequently used for PCR amplification using the specific primers for MMP-2, and GAPDH [22,53] using Taq DNA polymerase (Takra). PCR conditions were 95°C for 5 min followed by 95°C for 1 min, 54°C for 30 sec and 72°C for 30 sec for 30 cycles with a final extension for 5 min at 72°C. Ten μl of PCR product from each sample was subjected to electrophoresis on a 1.5% agarose gel. Gel was stained with ethidium bromide and destained.

### In vitro invasion assay

In vitro cellular invasion was assayed by determining the ability of cells to invade a synthetic basement membrane (Matrigel, BD Bioscience). Briefly, polycarbonate filters (8 μm pore size) were coated with Matrigel at a concentration of 1 μg/ml and placed in modified Boyden chamber. Conditioned medium was collected from HEK293 cells stably transfected with PTTG cDNA or pcDNA3.1 vector. HUVEC cells were suspended in serum free medium. A total of 2 × 10^5 ^cells were transferred to the top chamber. Conditioned medium with and without MMP-2-antibody was added to the lower chamber. The cells were incubated at 37°C and allowed to invade through Matrigel barrier for 22 h. Following incubation non-migrated cells in the upper chamber were removed with a clean cotton swab. Migrated cells through the Matrigel were stained with Eosin and Giemsa. Cells were counted and invasion index was calculated by dividing the percent of cells that migrated through the Matrigel by the percent of cells that moved through the pores of uncoated membrane.

### Wound migration assay

The wound migration assay was performed as described by Leavesley et al. [54] with slight modifications. HUVEC were grown in 35 mm culture dishes to 80% confluencey. A wound was formed using a 200 μl pipette tip to clear the cell monolayer, and the boundary of the wound was marked. Cells were then washed three times with PBS. The CM was applied in different combinations with and without MMP-2 blocking antibody. Mouse IgG (Sigma) was used as a control. Cells were incubated for 18–20 h at 37°C under 5% CO_2 _atmosphere. After incubation, cells were fixed with methanol, stained with Giemsa stain diluted 1:10 (Sigma Chemical Co.), and photographed. Cell migration was measured by counting the number of cells that migrated into the clear space using an Olympus microscope at 40X fitted with an ocular grid. The values represented are the mean of four different random fields. The fold change was determined by dividing the mean number of cells that moved from the wound edge to the wounded area in experimental culture by cells that moved from the wound edge in the control culture.

### Tubule formation assay in 3D matrigel matrix

To determine the effect of PTTG on tubule formation forty-eight well plates were coated with 20 μg/ml of Matrigel (GF-reduced BD Bedford MA). HUVEC (2 × 10^3 ^cell/ml) were plated in the Matrigel matrix with CM with or without MMP-2 blocking antibody. After 4 h, 100 μl of Matrigel-CM (Matrigel + conditioned medium mixed at a ratio of 1:2) was overlaid on cells. Cells were allowed to differentiate into tubule as described by Bootle-Wilbraham et al. [55]. After 3 days, tubule formation was assessed by fixing the cells with methanol for 15 min, followed by rinsing with PBS. Three random fields were viewed in triplicate wells for each test condition under high power Olympus (400X) microscope. Color images were captured using Kodak DC290 digital camera linked to a computer with Adobe Photoshop 7 software. Quantification of tubule formation was carried out by counting the number of tubule branched in the total area covered by tubules in each field using image analysis software Photoshop version 7. A numerical value was assigned to each pattern according to the network of tubule formation. A numerical value of 0–1 was assigned when the cells are well separated, 2–3 when cells begin to migrate and align to form tubes, and 4–5 when capillary tubes or closed polygons begin to form.

### Measurement of MMP-2 gene promoter activity

To examine if PTTG activate the transcription of MMP-2 gene, we determine the MMP-2 gene promoter activity. A human MMP-2 gene promoter sequence (1959 bp upstream of the transcription start site) cloned into luciferase reporter gene construct (pGL2) was obtained from Dr. Etty Beneveniste, University of Alabama at Birmingham, AL [56]. HEK293 cells were co-transfected with five hundred ng of the MMP-2 gene promoter construct or pGL2 construct with 500 ng of pcDNA3.1 or PTTG cDNA construct. Renilla-luciferase construct (pRenilla-Luc, Promega) 100 ng was used as an internal control as described above. After 48 h of transfection, cells were washed with PBS and lysed with 500 μl of 1X cell lysis buffer from Promega. Luciferase and renila luciferase activities were measured using dual substrate system from Promega in Zylux Femtometer FB12 luminometer. Luciferase activity for each sample was normalized to renila activity used as an internal control.

### Statistical analysis

Statistical comparison of data was carried out by the Student's t test (for single comparison) or by one-way ANOVA (for multiple comparisons). Probability of p < 0.05 determined from the two-sided test was considered significant. The statistical analysis was carried out by using SPSS 10.0 software.

## Conclusion

In summary, our results demonstrate that PTTG contributes to cell migration, invasion and angiogenesis by induction of MMP-2 secretion and expression. Blocking of MMP-2 function by using MMP-2-specific antibody decreases the cell migration and invasion induced by PTTG, suggesting for a existence of relationship between PTTG levels and MMP-2 expression and activity. Our results demonstrate that blocking or down regulation of PTTG expression in tumors overexpressing PTTG may result in suppression of tumor growth and metastasis, through the down regulation of MMP-2 expression and activity. Based on these results, we conclude that PTTG may serve a potential molecular target for cancer treatment.

## Authors' contributions

MTM carried out all the experiments, analysis of the data and drafted the manuscript. SSK participated in study design, planning of the experiments and editing of the manuscript. All authors read and approved the final manuscript.
